# The Virulence of *S. marcescens* Strains Isolated From Contaminated Blood Products Is Divergent in the *C. elegans* Infection Model

**DOI:** 10.3389/fgene.2021.667062

**Published:** 2021-06-10

**Authors:** Alexander Diamandas, Mikhail R. Razon, Sandra Ramirez-Arcos, Ann Karen C. Brassinga

**Affiliations:** ^1^Department of Microbiology, Faculty of Science, University of Manitoba, Winnipeg, MB, Canada; ^2^Centre for Innovation, Canadian Blood Services, Ottawa, ON, Canada; ^3^Department of Biochemistry, Microbiology, and Immunology, University of Ottawa, Ottawa, ON, Canada

**Keywords:** serratia macrescens, virulence, genomic analyses, platelet concentrates, *C. elegans*

## Abstract

Bacterial contamination of platelet concentrates (PCs) can occur during blood donation or PC processing, necessitating routine screening to identify contaminated products in efforts to prevent adverse transfusion reactions in recipient patients. *Serratia marcescens* is a common bacterial contaminant, and its resilient nature coupled with genetic promiscuity imbue this environmental bacterium with resistance to disinfectants and antibiotics enhancing bacterial virulence. In this study, we aim to understand adaptive survival mechanisms through genetic characterization of two *S. marcescens* strains, CBS11 and CBS12, isolated from PCs by Canadian Blood Services. Genomic analyses of the two strains indicated that CBS11 has one chromosome and one plasmid (pAM01), whereas CBS12 has no plasmids. Phylogenetic analyses show that CBS11 and CBS12 are non-clonal strains, with CBS11 clustering closely with clinical strain CAV1492 and less so with environmental strain PWN146, and CBS12 clustering with a clinical strain AR_0027. Interestingly, pAM01 was most closely related to PWN146p1, a plasmid found in *S. marcescens* PWN146 strain associated with pinewood nematode *Bursaphelenchus xylophilus.* Lastly, the genomic diversity of CBS11 and CBS12 was not reflected in the antibiotic resistance profiles as they were remarkably similar to one another, but was reflected in the virulence phenotypes assessed in the *Caenorhabditis elegans* nematode infection model, with CBS11 being more virulent then CBS12. Taken together, we suggest that *S. marcescens* environmental isolates that feature evolutionary diverse genomics are better equipped to adapt and thrive in varied environments, such as that of PCs, and therefore is as much of a concern as multi-drug resistance for human infection potential.

## Introduction

The Gram negative *Serratia marcescens* bacterium is an opportunistic pathogen found to be responsible for a wide range of diseases including ocular, uropathogenetic, respiratory, soft tissue and septic infections (Mahlen, 2011). Nominally an environmental soil bacterium, the ability of *S. marcescens* to easily adapt to shifting physicochemical conditions has enabled it to survive and thrive in diverse environments, such as disinfectant solutions, making it a frequent culprit of nosocomial infections (Sunenshine et al., 1996; Hervé et al., 2015). Additionally, *S. marcescens* has been found to be a bacterial contaminant of blood platelet concentrates (PCs) causing adverse transfusion reactions in recipient patients (Védy et al., 2009; Greco-Stewart et al., 2012). During blood collection from donors, *S. marcescens* contamination can occur from the venipuncture site despite prior disinfection of the donor’s skin, or from transient bacteremia in the donor’s blood, or by needle contact with aerosolized bacteria from the surrounding environment (Schrezenmeier et al., 2007; Greco-Stewart et al., 2012). Routine culture screening of PCs for bacterial contamination is done by established methods such as the BacT/ALERT system. Because screening is typically done during early platelet storage, levels of *S. marcescens* contamination may be below the threshold detection level, thereby resulting in false negatives (Schrezenmeier et al., 2007; Greco-Stewart et al., 2012). Additionally, detection may also be thwarted by the biofilm-forming capabilities of *S. marcescens* demonstrated by some, but not all, clinical isolates (Greco-Stewart et al., 2012). Biofilms are an aggregate of microorganisms encased within a polymeric matrix that facilitate attachment to surfaces aiding in both environmental persistence and subversion of detection or eradication methods (Flemming and Wingender, 2010). Thus, false negative results can result due to the ability of some bacterial species to form biofilm on the interior surface of PC bags, thereby effectively hiding from the screening process. In any event, should a false negative occur, the storage media (containing anticoagulants and glucose at neutral pH) and environment (constant agitation at 22 ± 2^o^C in oxygen-permeable plastic bags) are the ideal conditions to foster bacterial proliferation.

The ability of *S. marcescens* to adapt and survive in diverse environments can be attributed to a number of genetic factors. Chromosomally-encoded porins and efflux systems contribute to bacterial resilience with the former modulating cell envelope permeability in response to changing physicochemical conditions, and the latter facilitating the removal of antibiotics (Fernaández and Hancock, 2012). Production of virulence factors such as hemolysins, proteases, siderophores and lipopolysaccharides enhance infection potential once host access is gained (Kurz et al., 2003). Finally, the propensity of *S. marcescens* to acquire antibiotic resistance genes through horizontal gene transfer amplifies its persistence (Blair et al., 2015).

Here we report the genomic analyses of two unique strains of *S. marcescens*, CBS11 and CBS12, originally isolated from PCs by Canadian Blood Services through detection by routine BacT/ALERT screening (Greco-Stewart et al., 2012). Although both strains were verified to be biofilm-negative, CBS11 grew better than CBS12 in PCs prompting us to further investigate these strains. In our analyses, we utilized a variety of bioinformatic software tools to characterize these two strains, and to determine their relatedness to eight other known strains of *S. marcescens* whose sequences were obtained from the National Center for Biotechnology Information (NCBI) database. Based on our chromosome sequence analyses, we found that the two clinical isolates diverged from each other, with CBS11 clustering with clinical strain CAV1492 and environmental strain PWN146, and CBS12 clustering with environmental strain AR_0027, suggesting that CBS11 and CBS12 are unique strains of *S. marcescens*. As part of the CBS11 genome, plasmid pAM01 surprisingly shared identity with *S. marcescens* plasmid PWNM146p1 isolated from the pinewood nematode *Bursaphelenchus xylophilus.* To relate our genomic analyses to virulent phenotypes, we employed the well-established *Caenorhabditis elegans* nematode infection model. In comparison to the reference *S. marcescens* Db11 strain, CBS11 exhibits hypervirulence whereas CBS12 exhibits virulence in a similar fashion to Db11.

## Materials and Methods

### Bacterial and Nematode Strains, and Genomes

*S. marcescens* CBS11/2010 (CBS11) and CBS12 (CBS12/2010) were provided by Canadian Blood Services (Ottawa, Canada) (Greco-Stewart et al., 2012), and in-house *S. marcescens* Db11 strain was used as a reference control for inclusion in genomic analyses and *C. elegans* survival assays (Kurz et al., 2003; Iguchi et al., 2014). *C. elegans* N2 strain was thawed from frozen stock [S Buffer (129 mL 0.05 M K_2_HPO_4_, 871 mL 0.05 M KH_2_PO_4_, 5.85 g NaCl in 1 L) + 15% glycerol] and maintained at 15^o^C on Nematode Growth Medium (NGM) media spotted with live *E. coli* OP50 as a food source, and were manipulated using established techniques (Hope, 1999). All bacterial strains were grown on Luria Bertani (LB) broth or agar (Difco) at 37^o^C. Comparative genomic analyses were carried out on whole genome sequences of CBS11 and CBS12, along with eight *S. marcescens* strains (AR_0027, Db11, RSC-14, CAV1492, FDAARGOS_65, SM39, WW4, PWN146) retrieved from GenBank (NCBI). The genome of CBS11 included one chromosome and one plasmid pAM01, whereas the genome of CBS12 comprised one chromosome (Diamandas et al., 2020).

### Phylogenetic Analyses of CBS11 and CBS12

The phylogeny of the CBS11 and CBS12 chromosomes was analyzed using Geneious^TM^ software (2020.1.2 version) for alignment against whole genome sequences of eight other *S. marcescens* strains using the progressive Mauve alignment algorithm, which generates a number of Large Collinear Blocks (conserved segments that appear to be free of internal rearrangements), following methods described in Zhang and Lin (2012). Mauve is a multiple genome alignment system that uses an anchor selection algorithm to locate these LCBs with free end gaps (Darling et al., 2004). Of these LCBs generated for *S. marcescens* strains, the three with the longest sequences totaling 2,268,480 bp were concatenated together to build Neighbor-Joining consensus trees using the Tamura Nei genetic distance model, and bootstrap analysis values were produced from 100 replicates (Hall, 2013). A similar approach was done regarding the pAM01 plasmid, with the modification being that the chromosomes of three strains (Db11, CBS11, and CBS), along with the sequence of PWN146p1, were aligned against pAM01 generating a tree based on 4 LCBs (totaling 1,619,984 bp).

### Analyses for Horizontal Genetic Transfer

The DNA sequences of CBS11 chromosome, pAM01 plasmid, and CBS12 chromosome were uploaded to IslandViewer^1^, a web-based software tool that integrates four genetic island prediction systems (IslandPath-DIMOB, SIGI-HMM, IslandPick and Islander) to detect and label the genetic islands, if any (Bertelli et al., 2017). The platform then generates a computerized image of the genetic islands found in the DNA sequence, and color coordinates the islands based on the method of detection.

### Determination of Antimicrobial Resistance Profile

To assess the presence of genes associated with antimicrobial resistance, the DNA sequences of the CBS11 chromosome, pAM01 plasmid, and CBS12 chromosome were uploaded to The Comprehensive Antibiotic Resistance Database (CARD)^2^, a web-based software tool which provides curated reference sequences and SNPs organized via the Antibiotic Resistance Ontology (ARO) (Alcock et al., 2020). CARD will identify the resistance genes present in a sequence and compare them to its collection of known resistance determinants. This collection is integrated into the Resistance Gene Identifier (RGI) tool used for resistome prediction from genome sequences.

### Minimal Inhibitory Concentration (MIC) Assays

The twofold broth micro-dilution method was used to determine the MIC of antibiotics (amikacin, ampicillin, ceftriaxone, gentamicin and chloramphenicol) as outlined in Singh et al. (2020) with the exclusion of induction steps. Briefly, 3 mL of LB broth was inoculated with an isolated colony from freshly-streaked LB plates of CBS11 or CBS12, incubated overnight on a roller drum at 37^o^C, sub-cultured 1:100 (v/v) in fresh 3 mL LB broth, and incubated on a roller drum at 37^o^C until mid-log phase. Cells were pelleted, re-suspended in 0.85% NaCl to reach 0.5 McFarland density standard, diluted 1:50 (v/v) in Muller-Hinton broth (Becton-Dickinson) of which 50 μL was used to inoculate the first well of a serial dilution row in a 96-well microplate. Plates were incubated statically at 37^o^C for 18 h, after which the wells were monitored for bacterial growth, and the MIC was determined in accordance to the well that featured the lowest concentration of antibiotic along with no visible cell pellet. The MIC assay included two technical replicates, and repeated three times independently.

### *C. elegans* Assays

*C. elegans* nematode survival assays were performed as previously described (Powell and Ausubel, 2008). For each *S. marcescens* strain tested, approximately 30 nematodes were seeded at larval stage L4 onto triplicate NGM plates spotted with overnight-grown bacteria lawn, incubated at 25^o^C and monitored on a daily basis for survival. Nematodes were transferred onto fresh spotted assay plates to separate subjects from progeny. Nematodes were considered dead if by lack of pharyngeal pumping and non-responsiveness to touch with a platinum pick. Nematodes that crawled off the plate were censored from analysis. Nematode survival was calculated by the Kaplan-Meier method and differences were tested for significance using the log-rank test (GraphPad Prism version 7.0).

## Results

### Phylogenetic Tree Analyses

To determine the phylogenetic placement of CBS11 and CBS12, we followed methods previously described by Zhang and Lin (2012) that aimed to build phylogenetic trees of closely related species with genomes that can be largely aligned to one another. A challenge with building the phylogeny of close relatives is that conventional genes with low mutation rates may have too few SNPs to determine divergence at the strain level. Thus, the Mauve progressive algorithm method searches for large segments of DNA [referred to as Local Collinear Blocks (LCB)] which are highly conserved, consistent between genomes, and have few rearrangements (Darling et al., 2004).

**FIGURE 1:**
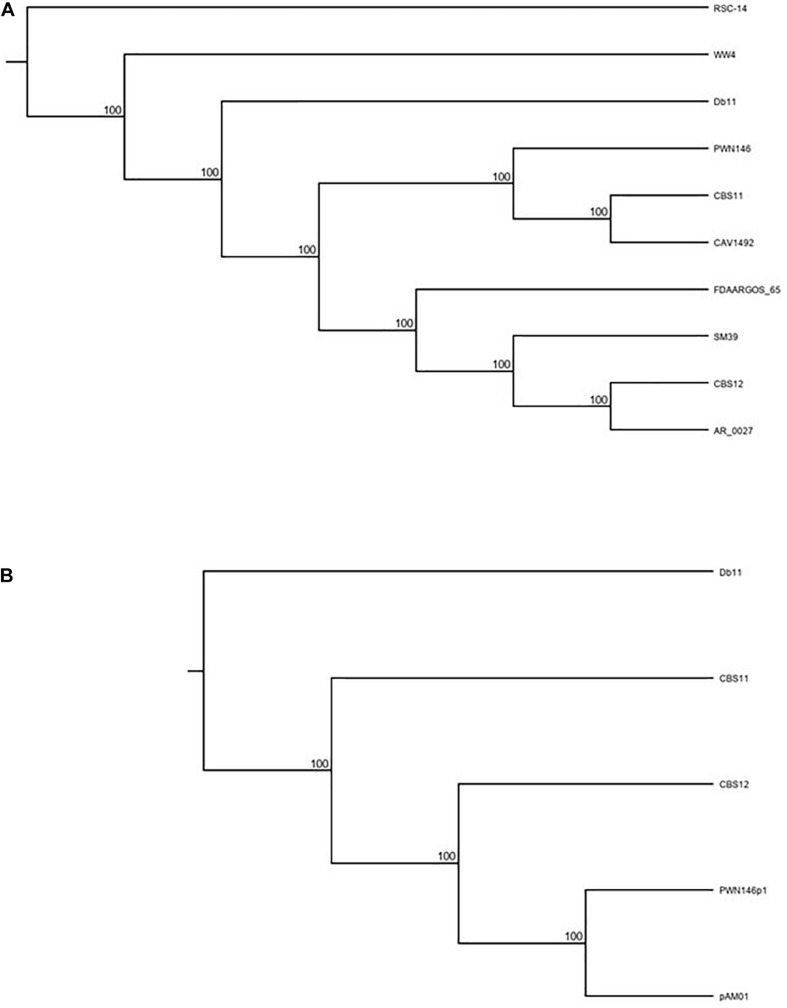
Tamura Nei Phylogenetic rooted tree (cladogram format) analyses of **(A)** CBS11 and CBS12 along with eight other known *S. marcescens* strains [RSC-14 (outgroup), Db11, SM39, CAV1492, WW4, FDAARGOS_65, AR_0027, PWN146]; and **(B)** pAM01 along with *S. marcescens* strains [CBS11, CBS12, Db11 (outgroup)] and *S. marcescens* plasmid PWN146p1. See text for further details.

Using this approach, we aligned the chromosomal sequences against a diverse range of eight *S. marcescens* strains using Geneious, a bioinformatics software platform commonly used for sequence analyses. Of the extracted LCBs, the three largest sequences were concatenated together and a Tamura-Nei phylogenetic tree was constructed which showed that both CBS11 and CBS12 grouped separately amongst the eight strains (Figure 1A and Table 1). CBS11 clustered with CAV1492, a KPC-producing nosocomial isolate from University of Virginia Health System, and a *Bursaphelenchus xylophilus* nematode isolate PWN146. CBS12 clustered with a CDC clinical isolate AR_0027 as a subgroup of a larger cluster that included clinical isolates SM39 and FDAARGOS_65 (Figure 1A and Table 1). Reference strain Db11, an insect pathogen isolate, clustered with environmental isolate WW4. Relating distantly, thereby serving as an outgroup in this tree, is environmental strain RCS-14, a plant-growth stimulating bacterium isolated from the roots of black nightshade *Solanum nigrum*. Taken together, the phylogenetic tree indicate that CBS11 and CBS12 are not clonal, but unique strains of *S. marcescens* (Figure 1A).

**TABLE 1:** Published *S. marcescens* genomes used for phylogenetic comparison.

Strain or plasmid	GenBank accession number	Isolation source (published reference)
AR_0027	NZ_CP026702.1	Clinical Isolate (none)
Db11	NZ_HG326223.1	Moribund fly (Flyg et al., 1980)
RSC–14	NZ_CP012639.1	*Solanum nigrum* roots (Khan et al., 2017)
CAV1492	NZ_CP011642.1	Clinical Isolate (Mathers et al., 2015)
FDAARGOS_65	NZ_CP026050.1	Clinical Isolate (none)
SM39	NZ_AP013063.1	Clinical isolate (Iguchi et al., 2014)
WW4	NC_020211.1	Paper machine (Chung et al., 2013)
PWN146	LT_575490.1	*B. xylophilus* nematode (Vicente et al., 2016)
PWN146p1	NZ_LT575491.1	*B. xylophilus* nematode (Vicente et al., 2016)

Whole plasmid BLAST search revealed that several large regions of pAM01 have high identity (>90%; data not shown) with a plasmid, PWN146p1, harbored by *S. marcescens* PWN146 (Table 1). To analyze the phylogenetic relatedness of pAM01 to PWN146p1, and to establish uniqueness of pAM01 from CBS11, a tree was constructed based on LCBs extracted from alignment of pAM01, PWN146p1, CBS11, CBS12, and Db11 sequences (Figure 1B). CBS12 served as a non-related reference strain, and distantly related Db11 served as an outgroup. Based on the generated tree, we can conclude that the pAM01 is genetically distinct from that of CBS11, and clusters with PWN146p1 corroborating the BLAST homology analysis.

### Genetic Islands of CBS11 and CBS12

The web-based software tool IslandViewer was used to analyze the chromosomes of CBS11 and CBS12, and pAM01 plasmid, to determine the presence of any genetic islands that may be introduced through horizontal genetic transfer. This tool utilizes four prediction systems to define various genetic islands, and the results are then color coordinated based on the method of detection. The chromosomes of both strains as well as the plasmid contain a variety of genetic islands (Supplementary Figure 1). Although there are no islands that are homologous between the two strains, these islands contain genes whose functional annotations are similar. Many of these genetic islands contain toxin secretion systems, phage components, transposases or integrases, and genetic replication or repair genes.

### Antimicrobial Resistance Profile

We utilized the CARD online software tool to locate the presence of antimicrobial resistance genes in the DNA sequences of CBS11, CBS12, and pAM01 plasmid (Supplementary Figure 2). CARD compares the uploaded sequences to its collection of known resistance determinants, then uses the RGI tool integrated into its software to assess the percentage (%) identity of resistance genes found in the uploaded sequences. The CARD software and RGI tool uses three different algorithms for AMR gene discovery: perfect, strict and loose. These three categories vary based on the accuracy of the genes’ identity to clinically confirmed AMR genes or mutations, with perfect hits being 100% identical, strict hits being genes with homology to known AMR genes, and loose hits being genes partially identical to known AMR genes, but contain differences that put them outside the predictive models used in the strict detection algorithm (Alcock et al., 2020). The results from the CARD database provided a total of 25 “strict” hits between CBS 11 and CBS 12, 847 “loose” hits, and 0 “perfect” hits (Table 2). Only one of the 25 “strict” genes was unique: the *tet(41)* gene in CBS 11 which confers tetracycline resistance. The % identity of these hits fell on a spectrum from between 20.53% to > 99%. Analysis of the pAM01 plasmid only returned one “loose” hit, a chloramphenicol phosphotransferase which only had a 26.06% identity despite sharing > 88% of its length with the CARD reference sequence. To correlate active antibiotic resistance with select identified hits, MIC assays were conducted for CBS11 and CBS12 in accordance to Clinical and Laboratory Standards Institute (CLSI, M100-27, 2017) guidelines for the following antibiotics: amikacin, ampicillin, ceftriaxone, gentamicin and chloramphenicol. With the exception for ampicillin in which CBS11 featured the MIC value of > 256 μg/mL as compared to the MIC value of 16 μg/mL for CBS12, both strains showed lack of resistance to antibiotics tested (data not shown). Taken together, CBS11 and CBS12 showed similar drug resistance profiles.

**TABLE 2:** Resistance gene identifier hits shared between CBS11 and CBS12 detected as “strict” and hits of pAM01 plasmid detected as “loose”.

RGI criteria	ARO term	SNP	Detection criteria	AMR gene family	Drug class	Resistance mechanism	% identity	% length
Strict	CRP		Protein homolog model	Resistance-nodulation-cell division (RND) antibiotic efflux pump	Macrolide antibiotic, fluoroquinolone antibiotic, penam	Antibiotic efflux	99.05	100.00
Strict	SRT-2		Protein homolog model	SRT beta-lactamase	Cephalosporin	Antibiotic inactivation	98.41	100.00
Strict	AAC(6′)-lc		Protein homolog model	AAC(6′)	Aminoglycoside antibiotic	Antibiotic inactivation	96.58	100.00
Strict	Escherichia coli EF-Tu mutants conferring resistance to Pulvomycin	R234F	Protein variant model	Elfamycin resistant EF-Tu	Elfamycin antibiotic	Antibiotic target alteration	95.17	96.33
Strict	Escherichia coli EF-Tu mutants conferring resistance to Protein homolog model Pulvomycin	R234F	Protein variant model	Elfamycin resistant EF-Tu	Elfamycin antibiotic	Antibiotic target alteration	95.17	96.33
Strict	Escherichia coli GlpT mutants conferring resistance to fosfomycin	E448K	Protein variant model	Antibiotic resistance GlpT	Fosfomycin	Antibiotic target alteration	90.87	99.56
Strict	Klebsiella pneumoniae KpnH		Protein homolog model	Major facilitator superfamily (MFS) antibiotic efflux pump	Macrolide antibiotic, fluoroquinolone antibiotic, aminoglycoside antibiotic, carbapenem, cephalosporin, penam, peptide antibiotic, penem	Antibiotic efflux	84.02	100.00
Strict	Klebsiella pneumoniae KpnF		Protein homolog model	Major facilitator superfamily (MFS) antibiotic efflux pump	Macrolide antibiotic, fluoroquinolone antibiotic, aminoglycoside antibiotic, carbapenem, cephalosporin, penam, peptide antibiotic, penem	Antibiotic efflux	73.39	100.00
Strict	adeF		Protein homolog model	Resistance-nodulation-cell division (RND) antibiotic efflux pump	Fluoroquinolone antibiotic, tetracycline antibiotic	Antibiotic efflux	60.1	98.87
Strict	Haemophilus influenzae PBP3 conferring resistance to beta-lactam antibiotics	D350N	Protein variant model	Penicillin-binding protein mutations conferring resistance to beta-lactam antibiotics	Cephalosporin, cephamycin, penam	Antibiotic target alteration	52.91	96.23
Strict	adeF		Protein homolog model	Resistance-nodulation-cell division (RND) antibiotic efflux pump	Fluoroquinolone antibiotic, tetracycline antibiotic	Antibiotic efflux	42.26	98.96
Strict	adeF		Protein homolog model	Resistance-nodulation-cell division (RND) antibiotic efflux pump	Fluoroquinolone antibiotic, tetracycline antibiotic	Antibiotic efflux	42.18	96.68
Loose	crmlv		Protein homolog model	Chloramphenicol phosphotransferase	Phenicol antibiotic	Antibiotic inactivation	26.06	88.76
Strict^a^	Tet(41)		Protein homolog model	Major facilitator superfamily (MFS) antibiotic efflux pump	Tetracycline antibiotic	Antibiotic efflux	9.49	98.98

**^*a*^Hits unique to CBS11.**

### CBS11 and CBS12 Virulence Determination

**FIGURE 2:**
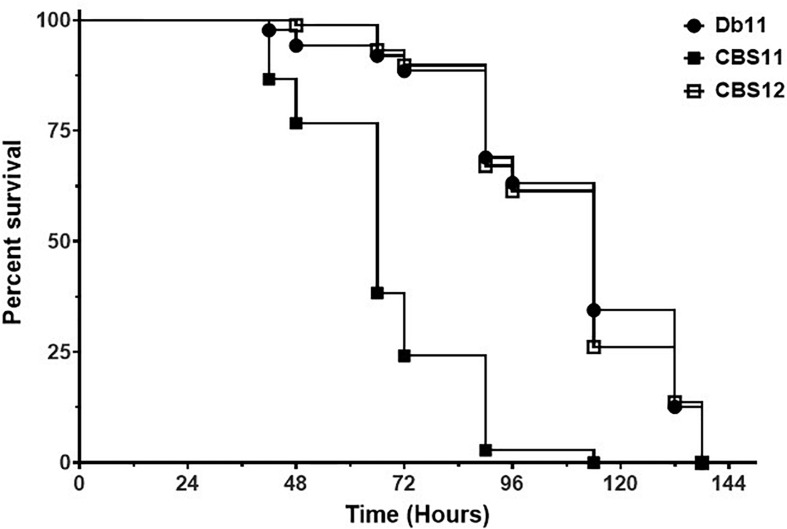
Survival of *C. elegans* on *S. marcescens* strains. Kaplan-Meier survival curve of *C. elegans* N2 nematodes fed Db11 (black circle, *n* = 90), CBS11 (black square, *n* = 90, *p* < 0.001), and CBS12 (open square, *n* = 90, *p* = 0.700). Survival curve is representative of one of three independent experiments.

*C. elegans* is a well-established tool for bacterial pathogenesis studies on a variety of human bacterial pathogens, and adept to screening of parental and isogenic mutant strains in a quick manner to correlate changes in virulence to genetic modifications (Sifri et al., 2005; Marsh and May, 2012). To assess the virulence properties of CBS11 and CBS12, the strains were implemented in *C. elegans* nematode survival assays along with Db11. The virulence and infection pathology of *S. marcescens* Db11 is well documented in *C. elegans* (Kurz et al., 2003), thereby serving as an excellent reference control. The generated Kaplein-Meier survival curve showed that Db11 shared similar virulent phenotypes achieving 50% population death by 120 h (5 days) fitting the reported parameters (Kurz et al., 2003). In comparison, CBS12 featured a survival curve similar to that of Db11, whereas CBS11 exhibited hyper-virulence reducing the period of time for 50% population death to 72 h (Figure 2).

## Discussion

*S. marcescens* is adept in surviving in a multitude of environments, and thus is a common bacterial contaminant of blood transfusion products (Greco-Stewart et al., 2012). In this study, we aimed to characterize two strains of *S. marcescens*, CBS11 and CBS12, in efforts to define the genetic basis of adaptive mechanisms enabling bacterial survival in PCs. The two strains were previously isolated by Canadian Blood Services through routine BacT/ALERT screening of PCs (Greco-Stewart et al., 2012). It was reported that the growth kinetic profiles of CBS11 and CBS12 were similar in LB broth, but different in PCs with the growth rate of CBS11 better than that of CBS12. While the physicochemical storage conditions of PCs (neutral pH, high glucose content, 20–24^o^C with agitation) are conducive for bacterial growth, PCs themselves have been shown to have moderately to extremely inhibitory effects on a variety of Gram positive and negative microorganisms (Drago et al., 2013; Varshney et al., 2019). Thus, stored PCs present a difficult growth environment for many different bacterial species due to the presence of immune factors such as antimicrobial peptides. Nevertheless, the ability of CBS11, but not CBS12, to thrive in PCs alludes to the possibility that CBS12 lack certain genetic features that would otherwise aid in its survival in diverse environments. Therefore, we utilized a variety of bioinformatic methods to aid in understanding the genetic features of CBS11 and CBS12, as well as assess the virulent properties of each strain using the *C. elegans* nematode infection model.

Phylogenetic placement can be informative in identifying lineages or ancestral ecological niches (Zhang and Lin, 2012). The alignment of CBS11 and CBS12 chromosomes against eight other completed *S. marcescens* genome sequences, chosen for the diversity in isolation sources (Table 1), showed that CBS11 and CBS12 are genetically distinct from each other. Adding to the uniqueness, a multitude of genetic islands are featured in CBS11, pAM01 and CBS12. Although no islands are homologous between the two strains, nor with the plasmid, many of the islands present in the three samples contain similar functional annotations. Since genetic islands are regions of DNA that contain evidence of horizontal genetic transfer (HGT) (Land et al., 2015), this suggests that CBS11 and CBS12 likely interact with a wide range of organisms, and therefore participate in horizontal gene transfer. Despite the genetic distinctiveness, similar drug resistance profiles were observed for CBS11 and CBS12 as determined by the CARD RGI tool (Table 2), and verified by MIC assays for select antibiotics.

In the *C. elegans* nematode infection model, CBS11 demonstrated hyper-virulence whereas CBS12 showed virulence similar to that of the reference Db11 strain. Frequently, virulence factors identified in *C. elegans* survival assays were found to be required for virulence in mammalian infection models. For instance, use of the *C. elegans* model in the screening of a Db11 transposon mutant library led to the identification of several hypo-virulent mutant strains that were also likewise hypo-virulent in a bronchial epithelial 16HBE14o- cell-line and murine infection models (Kurz et al., 2003). Recently, efforts have been made to define a core genome for multidrug resistance and virulence factors inherent for successful *S. marcescens* human infections. As a first report, the genome sequence of insect pathogen Db11 was compared to that of a human MDR clinical isolate SM39, isolated from a septicemic patient in Japan in 1999 (Iguchi et al., 2014). SM39 also harbors two plasmids, pSMC1 and pSMC2, whereas Db11 lacks plasmids. Nevertheless, chromosomally-encoded MDR factors were highly conserved between the two strains, but the proportion of shared virulence factors (excluding those carried on the plasmids) was small indicating the host-specific nature of each strain in accordance to its environmental niche. This host specificity is exemplified by the ability of SM39, but not Db11, to metabolize nitrate as a source of nitrogen; nitrate is present in urine, thus this metabolic capability favors the occurrence of urinary tract infections thereby establishing a successful human infection. Thus, in this view, the hyper-virulent phenotype of CBS11 is most likely due to the presence of pAM01, that shares high identity with that of PWN1946p1 that is part of the genome of *S. marcescens* PWN146, a microorganism associated with the pinewood nematode *B. xylophilius*. Genetic analyses of pAM01 are ongoing, but the plasmid is noted to encode a ShlB/FhaC/HecB hemolysin, which is a virulence factor for *C. elegans*, *Drosophila melangostar* and mice as reported in Kurz et al. (2003).

In summary, the data generated in this study could be used toward continuing efforts to prevent adverse transfusion reactions by improving PC storage conditions such that bacterial proliferation is minimized or eliminated. Furthermore, the genome sequences of CBS11 and CBS12 add to the growing repertoire of sequenced *S. marcescens* nosocomial and environmental isolates enabling further understanding of the genetic basis of virulence in connection to adaptive survival mechanisms and antimicrobial resistance. For example, a recent study conducted phylogenetic and comparative genomic analyses of 32 *S. marcescens* strains isolated from different ecological niches, and determined that pathogenic and environmental isolates intrinsically possessed equal number of antibiotic resistance genes, particularly those associated with efflux systems, generally acquired by HGT (Sandner-Miranda et al., 2018). Thus, the environment is the natural reservoir for HGT-mediated elements that when coupled with adaptive survival mechanisms that include efflux systems and porins, promote bacterial persistence and resilience, and in some cases, host-specificity.

## Data Availability Statement

The raw data supporting the conclusions of this article will be made available by the authors, without undue reservation.

## Author Contributions

AD and MR carried out the phylogenetic, genomic, and CARD analyses. AD did the *C. elegans* infection assays and wrote the initial draft of the manuscript. AB conceptualized and provided research funds for study undertaken and finalized manuscript draft. SR-A provided the *S. marcescens* strains and reviewed, edited, and final manuscript draft. All authors contributed to the article and approved the submitted version.

## Conflict of Interest

The authors declare that the research was conducted in the absence of any commercial or financial relationships that could be construed as a potential conflict of interest.

## Footnotes

1 http://www.pathogenomics.sfu.ca/islandviewer/

2 https://card.mcmaster.ca/

## Supplementary Material

Supplementary Figure 1 Genomic Islands of **(A)** CBS11, **(B)** CBS12, and **(C)** pAM01. The islands are marked according to the method of detection; SIGI-HMM (Orange), DIMOB (Blue), IslandPick (Green), and Integrated (Red).

Supplementary Figure 2 CARD Resistance Gene Identifier program hits in **(A)** CBS11, **(B)** CBS12, and **(C)** pAM01. The different levels of hits: Perfect (Green), Strict (Yellow), and Loose (Orange), are color coordinated.
