# Supplementary figures and images for: The Virulence of S. marcescens Strains Isolated From Contaminated Blood Products Is Divergent in the C. elegans Infection Model
Source: Front Genet. 2021 Jun 10;12:667062. doi: 10.3389/fgene.2021.667062 (PMC8222908; doi:10.3389/fgene.2021.667062)

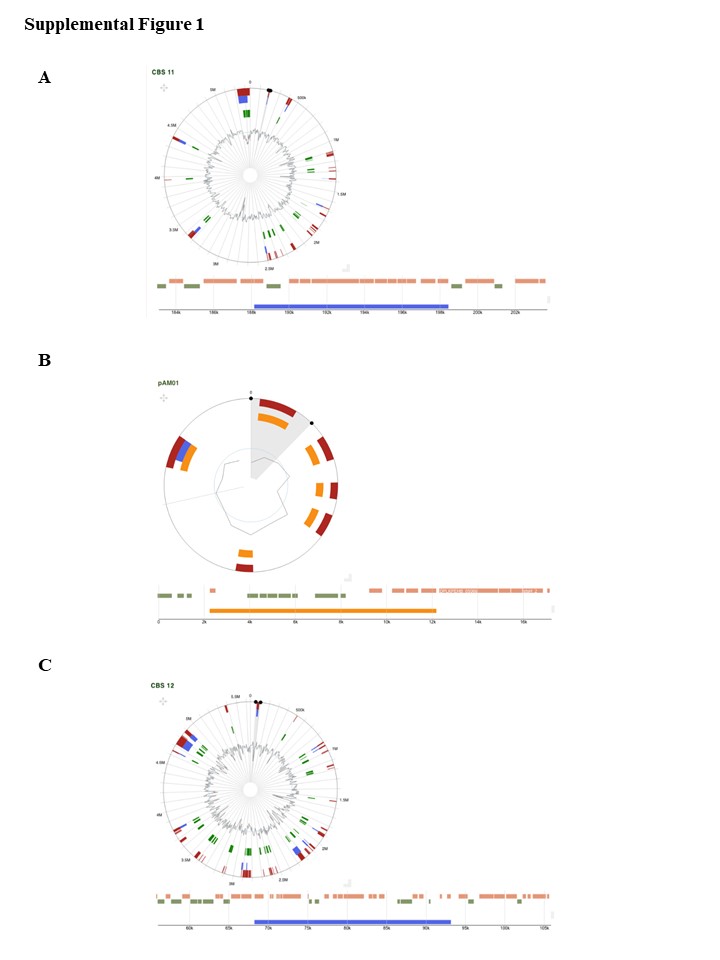

Supplement: Supplementary Figure 1 — Genomic Islands of (A) CBS11, (B) CBS12, and (C) pAM01. The islands are marked according to the method of detection; SIGI-HMM (Orange), DIMOB (Blue), IslandPick (Green), and Integrated (Red). [file Image_1.JPEG]

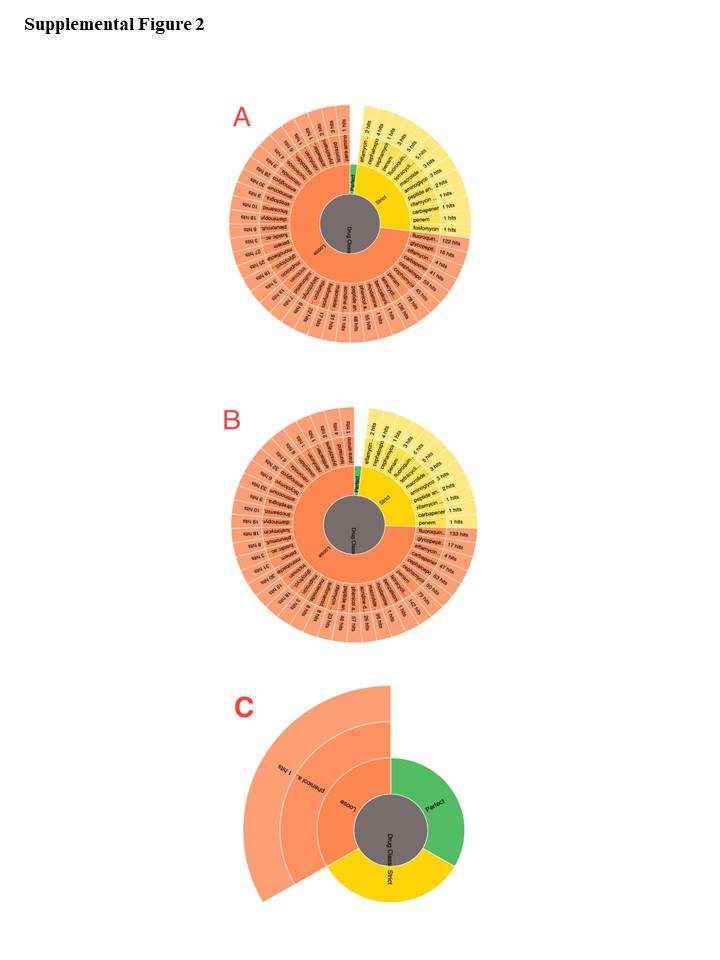

Supplement: Supplementary Figure 2 — CARD Resistance Gene Identifier program hits in (A) CBS11, (B) CBS12, and (C) pAM01. The different levels of hits: Perfect (Green), Strict (Yellow), and Loose (Orange), are color coordinated. [file Image_2.JPEG]
